# Why CEOs need advanced education and training for optimizing decisions in the development of innovative medicinal products? justification and recommendation

**DOI:** 10.3389/fphar.2024.1480338

**Published:** 2024-12-18

**Authors:** Peter-Jürgen Kramer, Gerd Bode, Rolf Bass

**Affiliations:** ^1^ Department of Chemistry, Technical University of Darmstadt, Darmstadt, Germany; ^2^ Institute for Pharmacology and Toxicology University of Goettingen, Göttingen, Germany; ^3^ Charité – Universitätsmedizin Berlin, Institute of Clinical Pharmacology and Toxicology, Berlin, Germany

**Keywords:** CEO and R&D strategies, training, decision making, research process, pharmatrain

## Abstract

Chief executives have been educated and trained how to handle business, to take executive decisions, and take care of financial and human resources in favor of the company they are leading. CEOs (chief executive officers) of innovative pharmaceutical businesses, among others, have only seldomly been trained to understand the immense time periods involved between decisions and their moment of impact, and the skills and languages used by their internal and external R&D (research and development) staff. R&D staff and regulators, however, have undergone full training, and are usually capable of understanding each other across their various specialties (among them compound finding, quality, safety, efficacy, efficiency, risk assessment and management). What is lacking is the specialized training of CEOs in sectors of R&D, which would benefit the company and patients alike. We propose that CEOs and upper management should undergo cross-border training in R&D topics–wherever possible across all sectors, but minimally to grasp such topics sufficiently to handle those scenarios demanding immediate decisions (be it the CEO cutting off developments, or R&D experts defending their continuation). Learning the language of regulators and R&D scientists will help CEOs to take better decisions. Training programs for R&D staff have been developed and implemented all over Europe and elsewhere. We propose to work with the PharmaTrain3.0 Syllabus (www.pharmatrain.eu/pharmatrain-syllabus) (for details see Supplementary Material), which would support clear-cut training of relevant topics by competent teaching staff towards certification of CEOs and high management. It is foreseen that understanding the language and comprehending the R&D issues and staff will help CEOs in achieving reasoned decisions. It is expected that such decisions will incorporate not only the reasons seen for discontinuation of R&D programs, but also those reasons, which favor their continuation under the same or different headlines (e.g., changing the initially proposed indication). Taken together, training according to “Good Training Practices” (GTP) will benefit the company and the patient, who will receive better medicines as early as possible.

## 1 Introduction

In the British journal “The Economist” there is a column on management which is named “Bartleby”. On 18 September 2023, Bartleby undertook an interesting thought exercise by asking: “*Who is the most important person in your company?*”

The article then stated: “*Questions are usually more interesting than answers. If you had to identify the most important person in your organization, there is an obvious answer, […]* “*the chief executive*”. *No cheese is bigger, no dog is more top. The most important decisions about the long-term direction of a company lie with the CEO; the hardest calls land on their desk; and the biggest pay cheques head their way. A board of directors might control their fate, but no one wields more power.*”

The search for an answer to the above question in a research-based pharmaceutical company might lead to a scientist who invented a new product, which became instrumental in securing the presence and future of the company. However, this would not be the usual answer!

In research-based pharmaceutical companies, the CEOs and those who have the ear of the boss on important issues (the so called “Panama Canal people”) are the most important people, having great decision-making power and long-term impact. They, therefore, have the power to take decisions about initiation, continuation or discontinuation of projects, thereby on short-, medium- and long-term research and development in the company. However, as a rule, these are marketing, business and financial experts. Beyond industry, no one would think that, for example, the commercial director of a university hospital or of a large research institute would have the final decision-making power over the various research and development projects. In the industry, however, this is normal. The CEO and the Executive Board determine the direction of a company, which in effect leads to management announcements such as:

“Our company plans to advance a more productive pipeline for new therapeutics using lessons gained from recent research setbacks with the guidance of a new management team. The goal is to accelerate decisions on high-risk, high-reward projects, because we need to deliver value fast, and that’s the mentality we”.

It is easy to conclude that both the participation of scientists in executive boards and a suitable training and qualification of CEOs and members of the executive board would be required to make research-based pharmaceutical companies more efficient and successful for achieving new innovative medicines and to optimize the huge financial investments in industrial drug R&D. In this paper we try to point out why this is crucial:

• What are the core competencies and soft skills of CEOs and executive board members in research-based pharmaceutical companies?
• How should top managers of research-based pharmaceutical companies be trained and educated top-down or bottom-up? Benefits for R&D staff?
• What might a certification course include in order to entitle managers to lead such a company? PharmaTrain Syllabus (v 3.0) already provides the required tools ready for adaptation to CEO training.

Our proposals on how to achieve optimization of R&D processes in pharmaceutical companies does not focus on the staff working in laboratories, clinics, project groups, controlling departments, etc., as it is usually done, when R&D processes are to be optimized. We focus on the role of CEOs and executive board members, currently and in future. We feel that this is an aspect that has not yet gained sufficient attention until now. CEOs and executive managers usually initiate the optimization of business processes in their organizations, and thus have an impact on research and development. They steer and define such processes but are not themselves subjected to the process (Horwitz N. B., 2018; Mintz, 2009). CEOs are usually not blamed for mistakes, and they often have no notion that a failure in the R&D departments could have been based on their decisions.

## 2 Pharmaceutical industry

### 2.1 Goal - Successful drugs

What is the goal of a pharmaceutical company? It is to be successful in marketing the most effective medicinal products. However, what is a successful drug? Which key criteria must be met?

Key criteria for a successful drug:

1. High medical need for an important therapeutic goal. Not today but in about 5 to 10 years!
2. Intellectual Property: patent protection for as long as possible and marketing authorization as early as possible. Investments made for such projects, but also for those projects that were carried out in parallel but failed, must flow back as income and must generate an overall profit for the company.
3. Innovative approach: accepted technology and scientifically accepted mechanism of action, which includes understanding the targeted disease.
4. Efficacy: Improved therapeutic efficacy for targeted medical indication.
5. Safety: Better benefit-risk ratio than existing pharmaceuticals.
6. Production: Favorable cost-benefit ratio, cost accepted by state health systems.
7. Regulatory approval: the new drug must meet the standards set by international guidelines and by competent authorities, such as the Food and Drug Administration (FDA) in the United States or the European Medicines Agency (EMA) in Europe.
8. Drug outcomes: “Efficiency” for the targeted indication under real-life conditions (Rosenkranz, 2024).

These points are well known, and numerous excellent experts have established optimized approaches in their R&D organizations. The overall success of the pharmaceutical industry, however, is modest. The vast majority of R&D projects in the pharmaceutical industry, despite huge financial and resource investments as well as continuous process optimization, cannot be successfully completed. Only a very small fraction of initiated projects turns out to be a real economic and medical success.

CEOs and board members are meant to be competent for the first two key criteria. There is no special thinking, language or training in place for e.g., EU and EFPIA to jointly them to handle most important issues, e.g., improvement of research, removing development bottlenecks in the drug development process, or (dis-) continuation of an R&D program–in a given case. R&D people, regulatory experts and specialists in synthesis, production, quality, safety, efficacy and (post-)marketing are expected to cover key criteria numbers 3 – 7, but also 1, 2 and 8. Specific trainings are available to them, and can be found as summarized in the PharmaTrain Syllabus 3.0 (see Supplementary Material). Such trainings include learning about thoughts, culture and language/thesaurus of colleagues, who have developed these processes separately.

The PharmaTrain Syllabus is one result of the first Innovative Medicines Initiative (created by the late Prof. Fritz Bühler), a joint project of the European Commission and EFPIA–guaranteeing participation of the best of the best for deriving best possible answers–case by case and in general. It was set out to fully grasp all developmental details of pharmaceutical R&D, laying out principles in a language common to all concerned. It encompasses 13 Sections starting with drug discovery and ending with health economics, modern outcomes research and issues on patient access (for further details see Supplementary Material).

### 2.2 Drug research and development (R&D) politics

It is often said that innovation cycles in medicine are constantly accelerating, however, this is only true for the applied technologies. The medical innovation, for example, the approval of new innovative medicinal drugs, lags behind. One example is Alzheimer’s disease, for which, despite enormous efforts, no scientific breakthrough, that would succeed in stopping or significantly slowing down the progress of the disease had been achieved for a long time. Contradictory decisions by FDA and EMA concerning lecanemab show that high expectations on efficacy and efficiency are in reality small and risky. Such are difficult to understand and accept by CEOs, R&D management, and patients concerned.

New software solutions, including simulation and automation technologies, appear frequently on the market and are always welcomed with much advance praise and high hopes. After some time, they are replaced by new procedures, usually, when it becomes clear that biological complexity prevented the intended solution, and the costs had increased enormously. As a matter of fact, the perceived complexity of biology does not decrease with new scientific discoveries but is often increased.

To optimize the R&D process, the management of most companies and the additionally committed consulting firms work intensively to be able to efficiently control the R&D process. To be able to control the R&D process efficiently, tools are required, which should allow a reliable measure of progress and its value. According to current management rules and those of frequently involved consultants, managers should be able to define which measures lead to success and which do not. For this purpose, so-called key performance indicators (KPIs) have been defined with which the success of measures can already be determined after a short time. The big question is which measurement parameters are meaningful regarding drug R&D. One of the principal problems measuring KPIs in pharma R&D is the very long period between the initial investments and the finished product, but there are also other principal problems, like the specific character of drug R&D projects in an area of tremendous biological complexity. From popular publications it was stressed that there is one exemption from this rule: mRNA-based vaccines for COVID-19. But as we know from the Nobel Prize in medicine in 2023 that Katalin Kariko and Drew Weismann had started their research 30 years ago: the toolbox needed was ready and available for use in developing anti-COVID vaccines almost on time. Such development will not happen soon again, even for those drug candidates using the same technology platform.

The very long period of development and the frequent data-driven changes in the R&D process make it difficult to measure the performance of an R&D organization in the way people are used to from other business and production areas. Drug R&D is one of the most technology-intensive industries, investing many billions of euros every year. It not only fails to deliver more success from investment but also lags behind the innovation performance of other industries. The current trend for new drug approvals shows an even further decrease.

**FIGURE 1:**
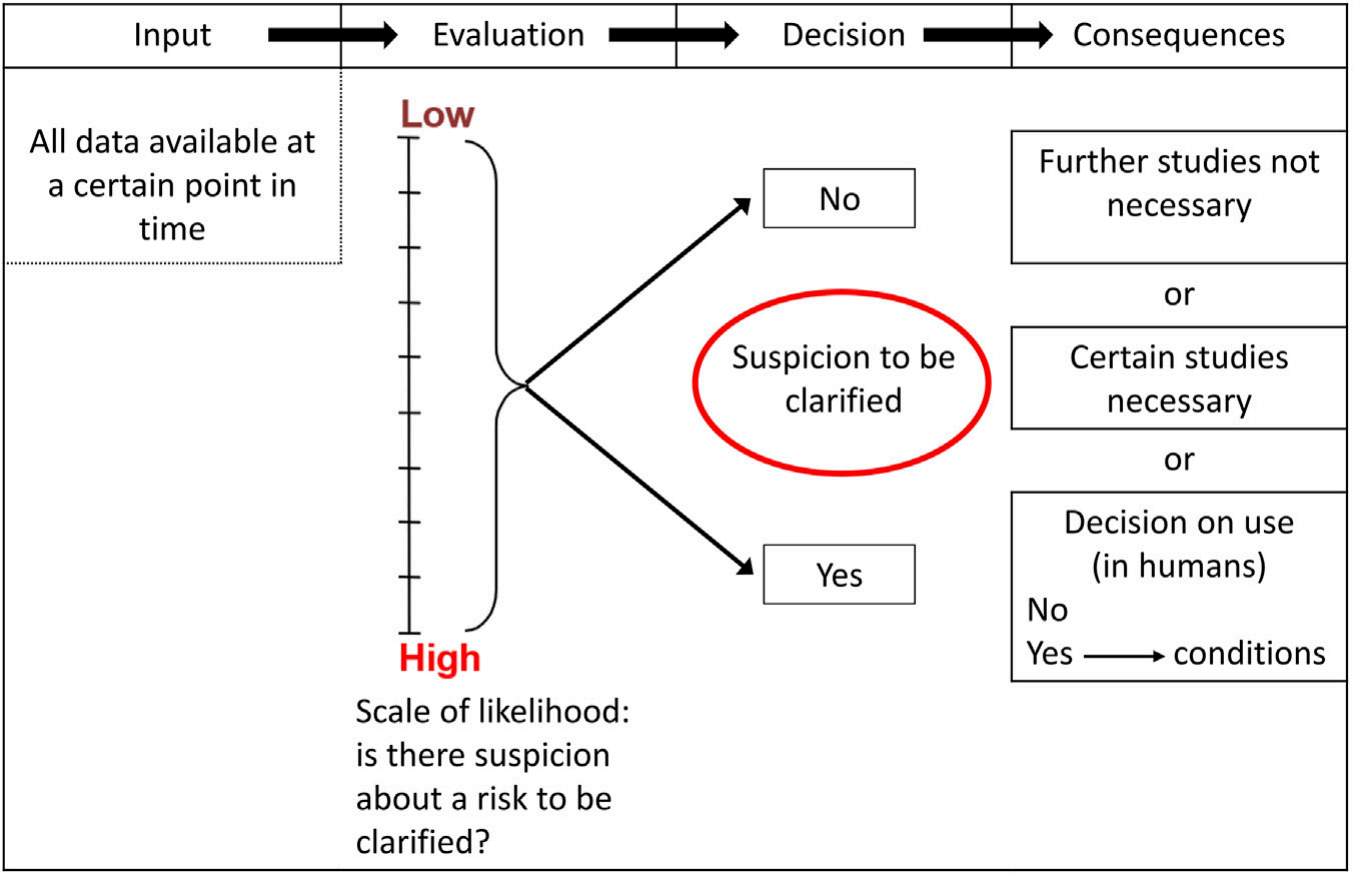
Sample flowchart on the development of preclinical strategies (Olejniczak et al., 2001).

Training of regulators and researchers is aimed especially at dealing with the frequent changes resulting from study outcomes, e.g., non-clinical and clinical results. The impact of missing or dangerous information in all subsequent steps as well as previous ones can be found in the PharmaTrain syllabus. Whilst CEOs and managers do not need to understand each and every detail, they should be capable of understanding and discussing single aspects as they arise case by case. Such aspects can be taken from the tables published by K. Olejniczak (Olejniczak et al., 2001). Here, CEOs, regulators and scientists can sit together to understand the need or not e.g., of reproductive toxicity testing at a certain stage of drug development derived from risk assessment and mitigation, and the possible impact on overall development–when such toxicity would be expected to possibly happen. It is the crux of medicines development that upper management decisions are often taken without understanding the real risk issues and their probabilities (Figure 1). There is no quick fix, but regulators and scientists should be alert for knowledge deficiencies of CEOs and upper management and urgently recommend special trainings. Again, the catalogue of PharmaTrain contains a complete compilation of potentially upcoming issues.

### 2.3 Drug research and development (R&D) management strategy

Managers and consulting companies try to take lessons learnt from more successful and faster industries to improve and accelerate the drug R&D process. According to common management theories, R&D functions must work closely with the commercial and corporate strategy teams to understand what the company wants to achieve. The R&D team should be guided by the priorities of the company and at the same time make clear what is scientifically and technically possible and feasible, today also including the use of artificial intelligence (AI) in clinical trials, pharmacovigilance, etc. The R&D strategy and corporate strategy must be aligned to answer questions such as: What are the company’s goals? Which R&D activities are required to achieve them?

Aligning the two above mentioned strategies is not easy. Usually in companies, the corporate strategy is set out prospectively in a five-year plan or covers even shorter periods. However, this is too short a time horizon to manage R&D in the pharma industry, where the product development period is much longer. To make this first step right, corporate strategy leaders should actively engage in research and development, which also requires a deep understanding of the relevant scientific background.

This happens at a time, when no details on go/no go decisions during later development are available. Therefore, corporate strategy leaders should have a basic understanding of R&D opportunities and potential pitfalls. Whereas the PharmaTrain syllabus encompasses all possible aspects, it cannot be expected that a full training will be taken by all CEOs and controlling managements. From our point of view such training is highly recommended, and will benefit in “developing better medicines faster” (slogan of PharmaTrain).

### 2.4 Drug research and development (R&D) technical prerequisites

An important prerequisite is digital empowerment, which today affects almost every aspect of the R&D function. Artificial intelligence can be used primarily in the early stages of projects to help identify new biological mechanisms or new applications of existing technologies. Based on this, automated screening procedures with high throughput can help to carry out a selection of possible candidates. Digital simulations can be used if no substances have yet been synthesized. Digital communication tools address the connectivity issues that occur with geographically distributed project teams, as is normal in large pharmaceutical companies. These tools have become indispensable for bringing together the often geographically distant employees in order to exploit synergies. However, truly innovative ideas often arise in direct personal encounters between people. The serendipity of chance encounters can be one of the conditions of many breakthrough innovations. Whether this can be generated significantly with so-called innovation centers has not been shown until now.

Most importantly, developing a strategy for pharmaceutical R&D involves some unique challenges that other industries do not face. On the one hand, scientists and regulatory employees have to take decisions that go far beyond their scientific or regulatory core competences, such as customer, market and economic factors–and these decisions may have consequences much longer than the 5-year business plan of the company. On the other hand, stakeholders, up to the senior managers, governed by company business criteria and residing outside the R&D laboratories, have to understand complex technologies and development processes and even more complex biological relationships and think about a much longer time horizon than they are accustomed to, before overriding the decisions of scientists and regulators.

General and specific needs and opportunities for training CEOs and senior managers can be taken from decision-tree risk strategies (Olejniczak et al., 2001), and from the PharmaTrain Syllabus v 3.0, 2024. It should be stressed that the need for training arises at specific and unforeseen decision points, calling for action and training by demand and on the spot. To avoid errors from fast trouble shooting, long-term full training is preferred.

### 2.5 Drug research and development (R&D) process

#### 2.5.1 Steering R&D processes

Among many other questions, the failure of the majority of R&D projects raises one big question: Why is steering drug R&D processes and projects so difficult?

Of course, this question is not new, and answers have already been taught by business schools or international consulting companies. Pharmaceutical companies invest considerably to learn from these organizations on how to improve and to increase the outcomes quantitatively and qualitatively. One basic problem answering this question and finding an effective solution is the available database. No company, neither in the case of success nor failure, honestly and transparently states what has finally led to success or failure. On the contrary, the reason behind a success is usually kept confidential inside the company and the true reason for a failure is kept obscured so that the company does not suffer any damage to its image. At the least, no systemic errors should become public. All companies are obliged to the latter, out of consideration of the shareholder value.

Nevertheless, there are a number of questions that need to be answered. A selection of such questions could be:

1. Which project organization is optimal for R&D projects in pharma?
2. Which procedures, processes and communications are needed?
3. Which mindsets are crucial at the different hierarchical levels?
4. Which qualifications of the people involved are needed or optimal?
5. Which type of controlling is needed for not obstructing sensitive innovation processes?
6. Which type of funding is needed for a learning process? R&D projects are learning processes, and any day can deliver unexpected data which may force a complete change of the plan!

#### 2.5.2 Failure of R&D processes

The fact that high-tech R&D projects may fail is well known in industry and in research institutions. The reasons are often similar: mostly insufficient communication with potential customers, over-optimistic targets and ballooning costs, resulting in the product not being sold on the market.

The advice given could be for example,: use an intelligent project management software and with this support, the projects will stay on track and will deliver on time and under budget. Successful projects begin with a good plan, but it may be hard to keep all necessary tasks under control. Many tasks may be dependent on others and therefore across the timeline. An IT tool may provide important help, e.g., by breaking the project into intelligent milestones. Such tools may be a decisive help for high-tech projects, and they may also be helpful for project managers in drug R&D, however, the problem with drug R&D is that a project involving biology is extremely complex.

A drug R&D project is a continuous learning project. The project team can go forward only step by step, adhering more or less to the straight initial outline of R&D. Every day results can be obtained which are, firstly, unexpected and, secondly, can cause a completely new situation. One question is always: is the organization’s strategy balanced between committed decisions and flexibility and learning? R&D strategies have very long-time horizons when it comes to real innovation and not just incremental innovation.

The development of a cancer drug can take more than 15 years and in the end the product has failed in oncology but may be approved for a completely different serious disease or may have proven to be a complete flop. In drug research and development, it is almost the rule that a therapeutic indication (project goal) has to be changed during the R&D process or proves to be completely impossible, e.g., because of side effects in the complex biological system that had not been predictable. A drug against depression or schizophrenia needs to consider that the human brain manages 10 trillion (10^13^) calculations in one second! The question to the researchers: Which one of those steps is going wrong and what happens to the neuronal network if one step is changed by a certain drug compound?

Prospectively, it is very important to understand that only very seldomly the therapeutic target indication on which a drug R&D project was started, is exactly that approved for the market. It, therefore, remains a central task of R&D departments, in the event of the failure of the original target indication, to try to extract a new therapeutic option for the project from the data accrued. This is important not only for financial, but also for scientific and ethical reasons because animals and humans treated up to this point with the drug candidate should not have been used in vain. The data already compiled is usually very valuable. Companies have been established which have specialized in developing successful drugs from failed projects of other companies (drug repurposing).

A number of top drug repurposing companies can be found here: https://www.ventureradar.com/keyword/drug%20repurposing.

In this context, it should also be pointed out that a fundamental difficulty of drug R&D is to predict which type of drug will have a therapeutic and economic success 15 years from now. It is not known today what will really be needed in 15 years, and secondly, which type of drug will make a splash in the pharmaceutical market. Marketing departments do not know this either, because they analyze the current market and determine which blockbuster product the company is missing on the day after tomorrow.

Based on current new findings, research departments are trying to anticipate the future and dream of developing successful or even blockbuster drugs. However, one must not forget that truly innovative projects require a change in thinking and therefore always experience effective internal opposition. In addition, doubters are usually viewed as very intelligent, while enthusiastic followers of an idea are quickly viewed as naive and gullible. Drug research remains a bet on the future, because despite the help of modern techniques, human beings have a fundamental problem: they cannot reliably predict the future and are not able to include all eventualities in planning a project where on any day anything may happen. Even the annual budget planning for R&D projects remains an attempt to pretend to have the next year of the drug R&D projects under control. When doubt proliferates it becomes dangerous for an innovative project, especially when it proliferates into the executive management.

Despite all this, executive leaders must devote significant funds to drug research and development (R&D) even though such investments are risky, their potential less visible to the stakeholders and the public compared to many other investments, and typically bear fruit only after the executive manager has already left the company. Executive managers are under great pressure.

Although most drug developments are terminated during clinical development or toxicology, this may also happen for non-scientific reasons. According to the rules of responsible management, it is important to stop an expected to fail project as quickly as possible, even if the developing researchers might vote to continue development.

#### 2.5.3 Leadership qualification for R&D based pharmaceutical companies

In order to achieve a more successful industrial development of innovative drugs, the top managers of the companies should specifically be trained and educated for their specific task. So far, only the scientists and managers directly involved in research are fully trained bottom-up to generate more successful R&D drug projects. This is still important, but not only purposeful, because an additional tailor-made training and education system should also be set up top-down for executive managers!

In the healthcare sector, especially in the field of prescription-only drugs, in contrast to the normal market economy, it is not the individual consumer (patient/organization) who decides on the purchase of a certain product and on the question of whether the product is allowed to enter the market at all and to remain there. In addition, enormous public funding is being used in healthcare. The higher these contributions rise, the more it must be expected that calls for a communitarisation of certain parts of the system, for example, the pharmaceutical industry, will become louder and louder. The pharmaceutical industry is traditionally seen as an industry that makes high profits at the expense of society.

Balancing R&D costs versus the economic benefit (also under the pressure of the stakeholders) needed to further development of next generations of medicines needs to be communicated well. Communitarisation as practiced previously in the Eastern bloc did not yield better results.

## 3 Consequences for executive management

### 3.1 Leadership in R&D-based pharmaceutical companies

#### 3.1.1 Core competencies and soft skills

Executives need excellent management skills and leadership capabilities, however, contrary to pharmaceutical companies in the generics field, executives in research-based pharmaceutical companies should have the mindset of real entrepreneurs. They should not just be business and marketing experts. Which means that they should primarily be proud of their competent employees (including researchers) and their excellent drug products and only secondarily of profit. If a company creates excellent drug products, profit comes automatically!

Regarding Pharma R&D projects, modern management tends to expect well-structured or even linear narratives as in high-tech projects. In other words, there is a belief that after a series of intense scientific challenges, the obstacles of a project may culminate, but in the end good management leads to a successful result. Therefore, in case of failure of R&D projects, the responsible management and the research groups involved are considered to be substandard.

Reality, however, is somewhat different and by no means corresponds to these presumed linear narratives. This means that a senior manager in pharmaceutical R&D can do everything right but will still be at the mercy of biological complexity, which is orders of magnitude higher than people can imagine. In other words, nature has to play along for success. The manager has to be guided by science, this knowledge should lead to increased imagination and openness for alternatives, but then more importantly, the manager should have the strength to stick to it, even if the situation does not look promising in the short term.

Executives should have their own vision of what the company wants to achieve to contribute to medicine and medical needs, and how they would define leadership, to lead their employees to commit to this goal.

Executives should be keen to learn what is seen differently by other people in the company, including the capability of self-criticism. Self-criticism and an open mind are basic requirements for scientists, therefore for an executive manager of a science-based company this should also be one important skill, which could be improved by further training options.

Executives should be keen to learn from national and international competent authorities as to their perception of what type of drug should be developed and which support these authorities could give during the research and development process. In other words, a real dialogue should occur on a high level and not just the formal scientific advice which is defined in the guidelines. Of course, the formal scientific advice remains key for the project groups to support a proper development of high-quality medicines which are effective and safe.

Executives should have the courage to be trendsetters instead of being managers that would always do what executives of competitor companies are doing. To do what executives of other companies are doing means to minimize their own personal risk. In other words, if a CEO makes the same mistake as any other CEO, he doesn’t make a mistake. On the other hand, if a CEO tells the shareholders and investors that he or she wants to do everything differently than the others, he or she takes a very high personal risk. Motto: whoever does what everyone does, risks nothing, even if it is possibly wrong. If the subordinate hierarchy levels duck and also agree, the impression of making the right decision is further reinforced and this would be the safest way to take a wrong decision.

Executives should not concentrate on cost control (controlling). They must create mutual trust regarding the use of company resources and should leave considerable freedom to their innovative coworkers in research and development.

Executives must give the scientist the feeling that they are really interested and engaged in their projects. This can be achieved by regular and in addition spontaneous communication. A show of emotion is allowed, e.g., empathy, but also respect for other opinions combined with self- and social awareness is necessary.

Executives of research-based pharmaceutical companies need to be prepared for undesirable news coming from the projects. They must know how to properly respond to such news.

Executives must also be able to inspire, otherwise they will not be able to raise funds.

When developing the R&D strategy and steering R&D projects, it is important to consider which competencies already exist in the company and which still need to be developed. For example, are there competent executive employees (including board members and CEO) with overarching skills and working methods that can develop and manage such an organization? Which new and important technologies could be acquired by buying another company?

#### 3.1.2 Top-down training and education of executive managers

As pointed out, short-term decisions of executive managers have far-reaching and long-lasting consequences not only for economic and marketing issues, but also for the companies’ R&D departments and projects! However, present mutual misunderstanding between R&D and top management is common as both sides are using a different language and thinking. The two sides are talking past each other, though both are making valid points.

Researchers, to be scientifically successful, cannot change their scientific language and thinking (“subject-matter expertise”). Therefore, top managers should learn and understand R&D people. Executive managers must understand the language and the thinking of R&D people because this is the only way to build up knowledge regarding the real status of the company’s R&D projects and to take informed decisions.

A new thinking is going to take place: in former times in pharma industry the focus was always on developing “blockbusters”; thousands of patients should be treated repeatedly for long times. The antineoplastic agent = Kymriah, developed by Novartis, is a personalized T- cell therapy. The antineoplastic function can be successful through a single infusion, and the treatment success holds a probability today of 80%. This treatment costs 475,000 dollars for one patient. Novartis decided that patients should only pay for this drug when the treatment is successful. This “outcome–based” principle is absolutely new. The concept is altogether revolutionary compared to our traditional thinking for strategies in drug development.

Executive board members should therefore be forced to review their thinking and complete a kind of executive education program tailored to their specific needs. Trainers could be still active or former executive experts from different companies and authorities that possess much experience regarding science and regulation but would no longer be pursuing a professional career (e.g., pensioner), as they must be independent from management.

To build up such knowledge, a suitable basis could be taken from the content as taught and tested in PharmaTrain, which is used all over Europe, e.g., in the master courses of MDRA (Bonn University) and EUDIPHARM (Lyon University). ECPM (University of Basel) has already trained - in the master course in Medicines Development CEOs of small and medium sized companies (Mollett et al., 2024). For a list see the Supplementary Material. The details to be offered for training CEOs and board members are given in the Supplementary Material. For discussion and decision making, the flow charts developed by Olejniczak (Olejniczak et al., 2001) present a very good opportunity to include all concerned in deriving at proper and defendable decisions.

#### 3.1.3 Certification

A certification which would specifically entitle and qualify managers to lead a research-based pharmaceutical company can be easily derived from PharmaTrain and the Syllabus developed there (for details see Supplementary Material). The Syllabus is applied by a number of universities for certification and university degrees (e.g., Basel, Budapest, Lyon), and is being used by the University of Bonn for their course - and the authors are part of the teaching staffs.

## 4 Discussion and conclusion

Many of so-called failed projects in drug R&D seem to fail because the decision to change the path of R&D is taken by the executive management. Such should be a good reason to reflect on possibilities to put this part of the process under a kind of quality check. In the usual quality assurance process, each and every person involved is checked on their level of qualification. The question always asked is: “Is the qualification level sufficiently high with regard to the task to be performed?”

As executive managers, including CEOs, take high level, short-term but long-lasting and far-reaching decisions also regarding the focus and fate of R&D, their qualification should also be high-level regarding understanding science and regulatory issues involved. In effect, corporate strategy leaders should actively engage in research and development, which also requires a deep understanding of the relevant scientific background, and the problems of risk-based decisions–before and after study results become available. In their own interest stakeholders and investors in the R&D based pharmaceutical industry should make sure that only those executive managers will lead the companies and make decisions on R&D that have proven to be highly qualified also in this specific sense.
