# Supplementary material for: Why CEOs need advanced education and training for optimizing decisions in the development of innovative medicinal products? justification and recommendation
Source: Front Pharmacol. 2024 Dec 18;15:1480338. doi: 10.3389/fphar.2024.1480338 (PMC11695935; doi:10.3389/fphar.2024.1480338)
Supplement: Supplementary file 1 [file DataSheet1.docx]

Supplementary Material

# Supplementary Material

## PharmaTrain Syllabus sections (version 3.0 – 2024)

| Section 1: | Drug Discovery |
| --- | --- |
| Section 2: | Development of Medicines: Planning |
| Section 3: | Non-Clinical Testing |
| Section 4: | Pharmaceutical Development and Manufacturing |
| Section 5: | Exploratory Development: Molecule to Proof of Concept |
| Section 6: | Confirmatory Development |
| Section 7: | Clinical Trials |
| Section 8: | Ethics and Legal Issues |
| Section 9: | Data Management and Statistics |
| Section 10: | Regulatory Affairs |
| Section 11: | Patient Safety, Pharmacovigilance and Pharmacoepidemiology |
| Section 12: | Medical Information, Education, Promotion: Communication Strategies and Channels |
| Section 13: | Health Economics, Outcomes Research, Pharmacoeconomics and Patient Access |

## Some samples setting out topics with specific relevance for CEO training

(Not mentioning financial planning and its implications, or interaction with regulatory authorities, not addressing marketing issues, and not-withstanding case by case importance and impact of other parts)

Section 1.2. Strategy, organization and rationalization of research including collaborations and co-development. The process of due diligence and in- and out-licensing

Section 2.3. Project management techniques: drug development plan, project teams, tools and decision-making from target product profile to registration dossier submission and lifecycle management.

Section 2.5. Pediatric development planning, a. for products indicated primarily for pediatric patients and b. for products developed for adults and requiring a pediatric investigation plan (PIP); orchestration of these parallel development

Section 3.6. The scheduling of non-clinical testing linked to product development plans, regulatory requirements, human and animal pharmacology, intended clinical use and routes of administration.

Section 3.8. The regular review of non-clinical testing, its inclusion into clinical trial protocols and investigator brochures. Relevance of expert interpretation and presentation of non-clinical testing results in the investigator brochure. Appropriate planning and correlation with the clinical evaluation of potential and observed toxic effects in patients.

Section 4.2. The economic primary production of new compounds and secondary production of research and market formulations

Section 5.1. Intended therapeutic indications and target product profile; biomarkers for target engagement, efficacy and safety end-points and criteria for ‘go’ / ‘no-go’ decisions for entry into humans and progression to proof-of-concept trials.

Section 5.2. Assessment of non-clinical data and the risk of hazards as prerequisites before administration to humans; including the calculation of starting and maximum dose for a trial.

Section 5.3. Phase 0 studies: exploratory microdose and subtherapeutic dose studies; the importance, limitations and uses of microdoses. Use of radio imaging for product distribution.

Section 5.7. First-in-human studies: patients and healthy volunteers; principles of proof-of-concept and dose-finding studies; starting dose and dose-escalation plan for first-in-human and early clinical studies, including applicability of pharmacokinetics to dosage regimen and study design in first-in-human studies and subsequent Phase II and Phase III clinical trials.

Section 5.8. Biomarker and analytical method validation

Section 5.10. Impact of results on planned therapeutic indications, predicted dosage schedules and drug delivery concepts / formulations; additional non-clinical requirements; reformulation studies; new pharmacology studies; risk-prediction algorithms to assess safety risks and enable development of risk-management approaches to be applied during continued development.

Section 6.1. Options for the clinical development plan; asset risk assessment and mitigation; schedules and decision points for the confirmatory clinical development program.

Section 6.2. Translation of the defined target product profile into the confirmatory clinical development program design; pivotal and other Phase III studies; selection of primary and secondary endpoints and comparators for Phase III clinical trials; final definition of therapeutic indications; risk minimization measures for research participants.

Section 7.1 Application of ICH Good Clinical Practice to clinical studies.

Section 7.9 Clinical trial management from sponsor and site perspective including risk identification and management

Section 7.14 Adverse event assessment and reporting; emergency coverage provision.

Section 7.17 Risk-based quality management in clinical trials: Quality by Design, quality manual; standard operating procedures; quality assurance and quality control; independent audits; inspections.

Section 8.5. Ethical considerations in pharmaceutical medicine, including non-clinical studies, database searches, medicines’ advertising, clinical trial participant contact, recruitment and reimbursement, use of social media, data protection, confidentiality and pharmacovigilance.

Section 9.12. Endpoints: endpoint types (continuous, binary / categorical, time-to-event, count), data transformation, primary and secondary and exploratory endpoints, dealing with multiplicity

Section 10.4. Developing a regulatory strategy to support the development lifecycle management of medicines, devices, combination products and diagnostics

Section 10.21. Regulation and procedures for early access to medicines; named patient supply, compassionate use, PRIME procedure (EU); ACTU procedure (France); early access to medicines scheme (EAMS, UK)

Section 11.3. The concept of a structured integrative benefit-risk framework; patient involvement in strategy and assessment of risk mitigation and management approaches throughout the product lifecycle; documentation and review of benefit-risk assessment and risk management activities.

Section 11.4. Collection of safety data in clinical trials including adverse events and adverse events of special interest. Approaches to, and planning for, data pooling across trials and analysis of safety in subpopulations. Safety data reporting requirements during clinical trials - serious adverse events, SUSARs, aggregate safety data reports. Safety implications of breaches of good clinical practice.

Section 11.5. The role of investigators, clinicians, study monitors, sponsors and manufacturers in the pre- and post-marketing phases to detect, assess and report adverse events and suspected adverse drug reactions; regulatory reporting requirements for individual cases and aggregate data safety reports such as DSURs and PBRERs in the pre- and post-marketing phases respectively.

Section 11.11. Safety signal management pre- and post-marketing, including detection and validation of signals, evaluation and categorization of risks.

Section 11.12. Pre- and post-authorization risk mitigation and management including issue and

Section 12.3. Publication strategy for clinical trials and clinical research studies: guidelines for selection of journals and good writing practices; guidelines for quality of production. Lay language summaries.

Section 13.6. Health technology assessment and market access at the international, national and local levels. Consideration for advanced therapeutic medicinal products. (Budget impact analysis, pricing strategy).

## Some Examples Useful for Training of CEOs

- Support for planning towards sourcing and securing sufficient available finance before commencement of a specific project in medical product R&D including allocation of funding and budgeting. Decision on degree of freedom for the development team
- Understand project management techniques tools to allow support of decision-making from target product profile to registration dossier submission and lifecycle management. Definition of points of interaction between CEO and development team, and joint decision on the (dis-) continuation of a specific project in medical R&D
- Understand requirements for product development in special populations (from paediatric to senescent) and when to initiate which studies understood necessary and when – based on special development criteria
- Understand the need to investigate and detect the mode of action and the pharmaco-/toxicokinetic properties of the specific substance in all species concerned
- Understand the preclinical development plan in all necessary aspects including go and no-go decisions (e.g. early on genotoxicity or QT prolongation, or suspicious organ toxicity, and later on reproductive toxicity or carcinogenic potential), and the need for continued interaction between preclinical and clinical expertise and outcomes. Pharmaceutical product development (namely drug substance and drug product) will need to be interspersed depending on Q&A across all parties. Understand the opportunity to challenge, discontinue or change the direction of product development based on single/multiple study outcomes
- Understand – most importantly – the data supporting “First-in-human investigations” and the decisions to step up increase of doses (Intended therapeutic indications and target product profile; biomarkers for target engagement, efficacy and safety end-points and criteria for ‘go’ / ‘no-go’ decisions for:
- entry into humans and progression to proof-of-concept trials versus time into such studies;
- confirming intended therapeutic indications and target product profile; need and availability of biomarkers for target engagement, development of efficacy and safety end-points
  - Understanding criteria for ‘go’ / ‘no-go’ decisions for entry into humans and progression to proof-of-concept trials
  - Understand the impact of the repetitive and updated compilation of all non-clinical testing, and its inclusion into clinical trial protocols and investigator brochures; understand the need for risk mitigation
  - Understand the specific needs of product development (Q, S, E) depending on the nature of the substance/product (be it chemical, biological or vaccine)
  - Understand the impact of and where of production and up-scaling including the decisions on drug formulation
  - Understand the overall clinical development plan (from first-in-human to proof-of-concept, dose finding and up to Phase 2 and initial and confirmatory Phase 3 studies; understand modelling and simulation, model-informed development, tolerability, metabolism, pharmacokinetics, pharmacodynamics and their cross-impact)
  - Understand the impact of results – as they come in - on planned therapeutic indications, predicted dosage schedules and drug delivery concepts / formulations; additional non-clinical requirements; reformulation studies; new pharmacology studies; risk-prediction algorithms to assess safety risks and enable development of risk-management approaches to be applied during continued development
  - Understand “Good Practices” throughout and their impact
  - Understand the need for compilation of any documentation, study result in internationally agreed standards (ICH plus national peculiarities: study reports, IB and protocols)

## Interaction at work

Within and beyond specialties

Creating and updating coordinated testing strategies (preclinical testing strategies given as example)

Starting Point: input all data available on that date

The aim is to deliver instructions for decision making based on scientific knowledge, by considering all available data (not only preclinical) and the scientific knowledge at that point in time, when a decision on a certain preclinical trial (as a precondition of clinical trials or use) is to be made. Is there a risk leading to a suspicion that requires scientific clarification. The evaluation of the degree of risk as being “low” or “high” can only lead to one of the following decisions:

- No, there is no suspicion requiring experimental clarification,

- Yes, there is a suspicion requiring experimental clarification

Any potential type of damage to humans will be settled this way – using special flow or charts covering all such areas.

Similarly flowcharts can be prepared for any other area of drug development, providing answers whether a certain study will be needed to support e.g. stability of a drug, safety in a certain clinical setting, or needed to support use in certain patient populations.

The advantage of preparing and using flowcharts towards testing strategies is to put things on the table, sit around the table and discuss the situation until common understanding of next developmental steps can be taken. Next steps will then be either to move forward ot once or to select proper studies to remedy the situation.

# Supplementary Figure


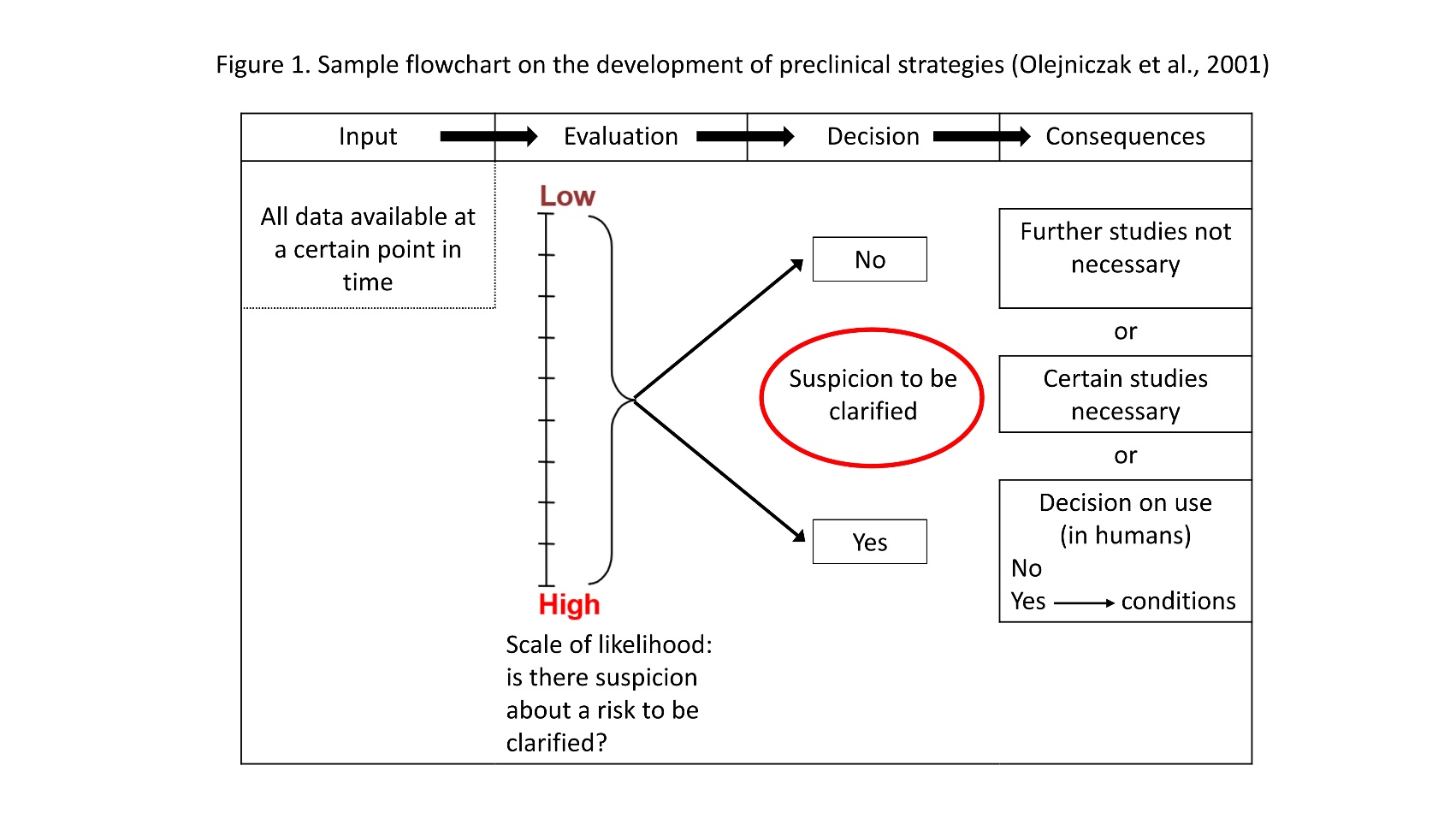


**Supplementary Figure 1.** Sample flowchart on the development of preclinical strategies (Olejniczak et al., 2001)
